# Achieving large and distant ancestral genome inference by using an improved discrete quantum-behaved particle swarm optimization algorithm

**DOI:** 10.1186/s12859-020-03833-7

**Published:** 2020-11-11

**Authors:** Zhaojuan Zhang, Wanliang Wang, Ruofan Xia, Gaofeng Pan, Jiandong Wang, Jijun Tang

**Affiliations:** 1 College of Computer Science and Technology, Zhejiang University of Technology, Liuhe Road, Hangzhou, China; 2 Department of Computer Science and Engineering, University of South Carolina, Assembly Street, Columbia, USA; 3 Tianjin Key Laboratory of Cognitive Computing and Application, Tianjin University, Yaguan Road, Tianjin, China

**Keywords:** Ancestral genome inference, Genome arrangement, DCJ sorting, Discrete optimization, Quantum-behaved particle swarm optimization

## Abstract

**Background:**

Reconstructing ancestral genomes is one of the central problems presented in genome rearrangement analysis since finding the most likely true ancestor is of significant importance in phylogenetic reconstruction. Large scale genome rearrangements can provide essential insights into evolutionary processes. However, when the genomes are large and distant, classical median solvers have failed to adequately address these challenges due to the exponential increase of the search space. Consequently, solving ancestral genome inference problems constitutes a task of paramount importance that continues to challenge the current methods used in this area, whose difficulty is further increased by the ongoing rapid accumulation of whole-genome data.

**Results:**

In response to these challenges, we provide two contributions for ancestral genome inference. First, an improved discrete quantum-behaved particle swarm optimization algorithm (IDQPSO) by averaging two of the fitness values is proposed to address the discrete search space. Second, we incorporate DCJ sorting into the IDQPSO (IDQPSO-Median). In comparison with the other methods, when the genomes are large and distant, IDQPSO-Median has the lowest median score, the highest adjacency accuracy, and the closest distance to the true ancestor. In addition, we have integrated our IDQPSO-Median approach with the GRAPPA framework. Our experiments show that this new phylogenetic method is very accurate and effective by using IDQPSO-Median.

**Conclusions:**

Our experimental results demonstrate the advantages of IDQPSO-Median approach over the other methods when the genomes are large and distant. When our experimental results are evaluated in a comprehensive manner, it is clear that the IDQPSO-Median approach we propose achieves better scalability compared to existing algorithms. Moreover, our experimental results by using simulated and real datasets confirm that the IDQPSO-Median, when integrated with the GRAPPA framework, outperforms other heuristics in terms of accuracy, while also continuing to infer phylogenies that were equivalent or close to the true trees within 5 days of computation, which is far beyond the difficulty level that can be handled by GRAPPA.

## Background

### Introduction

Advancements in the reconstruction of ancestral genomes constitute significant developments in Bioinformatics. Inferred ancestral genomes can be used to reconstruct deep evolutionary histories, which in turn, have been successfully applied to a wide range of problems facing contemporary society, including in controlling hereditary diseases, providing a better understanding of complex disease mechanisms for which effective detection and diagnostic pharmaceuticals drugs have then been developed, and inferring epidemic contact structure, among others [1–5]. With the advent of new sequencing techniques in conjunction with sophisticated computing technologies, the availability of whole genome sequences continues to increase. Critically, genome rearrangements are utilized in the analysis of whole genomes, and notwithstanding their rarity, genome rearrangements have provided invaluable information that has served to facilitate the solutions to the problems of key importance in biology and that has further deepened our fundamental understanding of biology itself.

As the smallest unrooted phylogenetic tree is defined by three leaves, one of the most significant problems in genome rearrangement analysis is termed as the Median Problem, which is defined as follows: given three input leaf genomes, find the genome (median) that minimizes the sum of evolutionary distances between the median and the three input genomes. Despite that the Median Problem is NP-hard for most criteria [6, 7], finding a solution for the median problem is essential since the recovered median is not only considered to be a good option for the ancestral genome but can further be used in a multitude of purposes in phylogenetic tree reconstructions.

To date, many studies which include the exact and heuristics methods have been developed to solve the median problem. Xu et al. proposed a branch-and-bound method using adequate subgraphs to decompose the median problem (ASMedian) [8, 9]. Then, in the GASTS package, the ASMedian approach was extended to reconstruct phylogenies with more than three genomes by minimizing the sum of the pairwise genomic distances between tree nodes [10]. Although ASMedian achieves excellent marks in the performance metrics of speed and accuracy, its feasible deployment is limited to small-scale datasets. Thus, Feijao et al. developed an algorithmic approach for ancestral reconstruction of gene orders using the concept of intermediate genomes, which obtained a better reconstruction of the true ancestral genome [11]. Feijão et al. later presented a closed equation for the single cut join (SCJ) distance model that accounts for duplications [12] and thereafter, introduced an integer linear program to solve the Median Problem but in a context where gene duplication events were now considered [13].

In addition to these types of heuristic-based models, metaheuristic algorithms are other effective methods for tackling complex optimization problems that has also been successfully incorporated into phylogenetics in order to solve various NP-hard problems. In 2005, Hill [14] developed a phylogenetic reconstruction tool for heuristic searching using the character-based genetic algorithm. However, it was not until 2013 that Gao et al. first proposed an approach to the Median Problem that used a genetic algorithm (GA) as its foundation and was further based on genomic sorting (GAMedian) in order to find the best sequence of evolutionary events to transform one genome into the other [15]. Gao et al. [16] next adopted a cooperative co-evolutionary genetic algorithm (CCGA) that divides a phylogenetic reconstruction problem into smaller sub-problems in order to infer ancestral genomes, which results consistently reflect more accurate reconstructions. Likewise, in an effort to overcome these limitations, Xia et al. [17] proposed a metaheuristic method that implements the simulated annealing median algorithm (SAMedian) to solve the Median Problem, albeit slightly less accurate, but with greater efficiency by producing these results with lower computational costs.

Existing methods utilized in reconstructing ancestral genomes also confront difficulties as a consequence of the failure of traditional optimization methods to address the complexities encountered in the practical applications in which they are deployed. In this vein, in the context of large and distant genomes, the problem of inferring ancestral genomes by reconstructing their ancestral sequences presents a highly-complex, multidimensional problem, whereby the domain constituting the inference search space although discrete, is also extremely large and is composed of high dimensional data. As a result, the process of inferring ancestral genomes continues to challenge the current optimization methods used in this area, which are further compounded by the continuing rapid accumulation of whole-genome data. Accordingly, achieving ancestral genomes with greater accuracy and increased computational feasibility in the context of limited memory space is vital to the ability to reconstruct ancestral genomes and more generally, for the continued advancement of the phylogenetic reconstruction discipline.

New algorithms of significance continue to be proposed, including algorithms using novel metaheuristic methods. Indeed, one of the most important metaheuristic algorithms, the quantum-behaved particle swarm optimization algorithm (QPSO) [18], was introduced by Sun et al. First, in order to overcome the shortcoming that QPSO is not applied to discrete space, an improved discrete QPSO algorithm (IDQPSO) that is inspired by adopting two averages of the fitness values is proposed. Second, the DCJ sorting is incorporated into the IDQPSO to address the median problem (IDQPSO-Median) since QPSO can have a better search capability in large-scale optimization problems. Furthermore, extensive experiments on simulated datasets and real datasets were conducted to evaluate the performance of IDQPSO-Median.

### Genome rearrangement events

Given a genome with one or more chromosomes, each chromosome can be labeled by a gene order to represent the direction and the relative positions of genes, represented as integers such as $\{g_1, g_2, \ldots , g_n \}$, each represents a homologous gene. Moreover, each gene is assigned an orientation that is either positive or negative, correspondingly denoted by $g_i$ or $-g_i$, respectively. The head and tail of a gene, $g_{i}$, are denoted by $g_i^h$ and $g_i^t$, respectively. If a gene is associated with a positive sign, then this means that the direction in which the gene should be read is from head to tail $(g_i^h \rightarrow g_i^t)$, whereas a negative sign associated with a gene indicates it should be read from tail to head $(g_i^t \rightarrow g_i^h)$. After considering the direction of the genes in each segment of a genome, a chromosome can be considered as an ordered set of oriented genes and then can also be classified as linear, meaning it is a sequence that has two ends, called telomeres (as defined below), or is circular, meaning it is a a sequence for which its head meets its tail. In this paper, we assume that each genome has the same number of genes and each gene appears exactly once.

Genome rearrangement events include the inversion (reversal), transposition, fusion, fission, and translocation operations. Assuming a genome is a signed gene order, denoted by $\{g_1,g_2,\ldots ,g_{i},\ldots ,g_{j},\ldots ,g_{n}\}$, then after a reversal operation, the genome is transformed to $\{g_1,g_2,\ldots ,g_{i-1},-g_{j},-g_{j-1},\ldots ,-g_{i},g_{j+1},\ldots ,g_{n}\}$. Further, assuming that $j < k$ and given three gene orders, denoted by $g_{i}$, $g_{j}$, and $g_{k}$, then a transposition operation can generate a new genome, which resulting genome is denoted by $\{g_1,g_2,\ldots ,g_{i-1},g_{j+1},\ldots ,g_{k-1},g_{i},\ldots ,g_{j},g_{k},\ldots ,g_{n}\}$. Given two genomes, the translocation operation is defined as occurring when the end of one chromosome is broken and then attached to the end of another chromosome, whereby in essence, two chromosomes exchange their ends. Meanwhile, the fission operation occurs when one chromosome splits into two chromosomes, while the fusion operation concatenates two chromosomes into one chromosome.

If $g_{i}$ is followed by $g_{j}$, or if $g_{j}$ is followed by $g_{i}$, then $g_{i}$ and $g_{j}$ are defined as adjacent. In addition, the adjacency of two consecutive genes can have the following four types: $\{g_i^h,g_j^h \},\{g_i^h,g_j^t \},\{g_i^t,g_j^h \}, \{g_i^t,g_j^t \}$. Correspondingly, a breakpoint occurs when two genes are adjacent in one genome but not in another genome. In the same vein, a telomere is defined as an extremity that is not adjacent to any other genes and is represented by a singleton set. Accordingly, in the context of this model, a genome is a set of adjacencies and telomeres such that the head or tail of any gene appears in exactly one adjacency or telomere.

### DCJ distance and DCJ sorting

The DCJ operation as proposed by Yancopoulos [19], not only adopts the universal DCJ operation but also goes further by considering all of the rearrangement events described above. The following four cases generally describe the sequence of DCJ operations in transforming one genome to the other:

- A pair of adjacencies $\{g_1,g_2 \}$ and $\{g_3,g_4 \}$ can be rejoined by the two adjacencies: $\{g_1,g_3 \}$ and $\{g_2,g_4 \}$ or $\{g_1,g_4 \}$ and $\{g_2,g_3 \}$.
- An adjacency $\{g_1,g_2 \}$ and a telomere $\{\circ \}$ can be rejoined as follows: by an adjacency $\{g_1,\circ \}$ and the telomere of $\{g_2 \}$ or in the alternative, by an adjacency $\{g_2,\circ \}$ and the telomere of $\{g_1\}$.
- A pair of telomeres $\{\circ \}$ and $\{\circ \}$ can be joined by the following adjacency: $\{\circ ,\circ \}$.
- An adjacency $\{g_1,g_2 \}$ can be cut by a telomere, thereby resulting in $\{g_1 \}$ and $\{g_2 \}$.

**Fig. 1:**
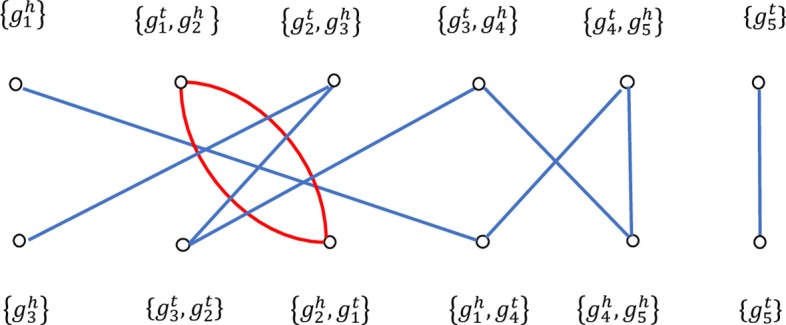
Adjacency graph of two genomes. Given $G_1=\{g_1,g_2,g_3,g_4,g_5 \}$ and $G_2=\{g_3,-g_2,-g_1,-g_4,g_5 \}$. The number of paths is 2, the number of cycles is 1, and the length of the genome is 5. By Eq (1), the DCJ distance between $G_1$ and $G_2$ is $n-(C+I/2) =3$

An adjacency graph (Fig. 1) is reconstructed with the adjacencies and telomeres in order to find the sequence of DCJ operations, which constitutes the DCJ distance (as defined below). As used in this model, the DCJ distance is defined as the number of DCJ operations required to transform one genome into the other. The DCJ distance between $G_1$ and $G_2$, denoted by $d_{DCJ}(G_1,G_2 )$, is calculated by

1 $$\begin{aligned} d_{DCJ}(G_1,G_2 )=n-(C+I/2) \end{aligned}$$

where *n* is the length of the genome, *C* is the number of cycles, and *I* is the number of odd edge paths. In the adjacency graph, a DCJ operation is optimal when it increases the number of cycles by one or the number of odd paths by two, such an optimal path reflected by the application of the DCJ operations can increase the sum of *C* and *I*/2 by one and still make $G_1$ one step closer to $G_2$.

Given two genomes $G_i$ and $G_j$, many divergent series of sequences of DCJ operations exist that can evolve one genome into the other. Differences among DCJ operations can change the number of odd paths and circles and typically also influence the structure of the common gene adjacencies presented. The DCJ sorting is defined as finding the sequence of DCJ events that transform on genome into another by using the minimum number of events. The evolutionary cost may be minimized if the median genome is situated on the sorting path created by the DCJ operations in transforming from one leaf (known) genome into the other. Two approaches have now been introduced that have expanded upon the DCJ operations framework first proposed by Yancopoulos by allowing for sampling of the sorting sequences comprising the solutions space produced by the DCJ operations: the first approach applies the greedy-sampling method by iteratively applying the DCJ operation in order to create a new adjacency graph [20], and the second approach uses a general-sampling method that considers and evaluates all of the cycle-splitting operations in identifying the structures of the compared genomes that cause the increase in the number of solutions with respect to a given lower bound [21].

### DCJ median problem

**Fig. 2:**
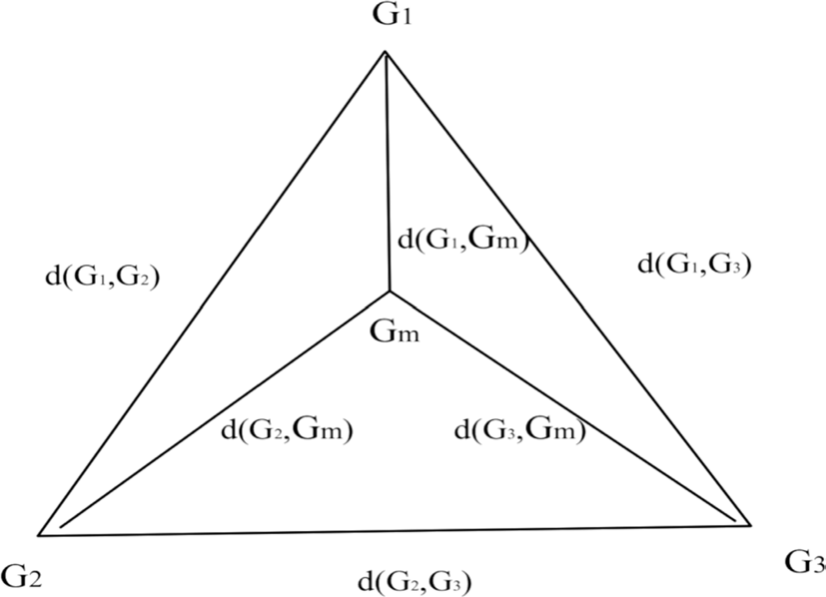
The median problem. Find a median genome $G_m$ that minimizes the sum of DCJ distance denoted by $\sum _{i=1}^{3}d(G_i,G_m)$ if given three genomes $G_1$, $G_2$, and $G_3$

The DCJ Median Problem is to find a median genome that minimizes the sum of DCJ Distances from the median genome to the three-leaf genomes. As illustrated in Fig. 2, the median genome for a solution of the DCJ Median Problem is considered to constitute a good option for an ancestral genome if it minimizes the evolutionary cost. The median score used for calculating the DCJ Median is given by

2 $$\begin{aligned} S_3=d(G_1,G_m )+d(G_2,G_m )+d(G_3,G_m ) \end{aligned}$$

where the DCJ distance between the median genome and each of three given genomes is denoted by *d*, the sum of all the DCJ distances is defined as $S_3$, the median genome is denoted by $G_m$, and the three given genomes are denoted by $G_1$, $G_2$ and $G_3$, respectively.

## Methods

### Quantum-behaved particle swarm optimization (QPSO)

Quantum-behaved particle swarm optimization is a stochastic searching algorithm that was inspired by the movement of particles in quantum space and was first proposed by Sun et al. [18]. QPSO is a population-based evolutionary computation algorithm, wherein each particle is considered as a candidate solution. The behavior of all particles is described by the quantum mechanics presented in the quantum time-space framework. According to the principles of quantum theory, the behavior of (and thus, the search process over) all particles is directed by the quantum-mechanical rules rather than the classical rules of Newtonian random motion. Because of the inherent uncertainty in any particle’s motion and attendant locations at successive times, the quantum state of each particle can appear anywhere in the search space.

Compared with other previously introduced evolutionary algorithms, the performance of QPSO achieves better results insofar as the performance indicator of global search capabilities are concerned. Because QPSO’s search covers the entire search space for each generation, it also increases the diversity of the population and thus, overcomes the problem of premature convergence currently existing in continuous optimization problems in this space. Furthermore, QPSO has been demonstrated to successfully solve a wide range of continuous optimization problems, including problems in multilevel thresholding image segmentation [22], network clustering [23], and optimal design [24], as well as in the strongly NP-hard combinatorial optimization problem of the multidimensional knapsack problem [25].

Based on the quantum physics theory, the quantum state of a particle represents its momentum and energy. Accordingly, the dynamic behavior of each particle is described by the wave function $\psi$. The normalized wave function is given by

3 $$\begin{aligned} \psi (y)=\frac{1}{\sqrt{L}}e^{-\left| y\right| / L } \end{aligned}$$

where *y* is set as $y=X-P_{id}$. *X* represents the current position, and $P_{id}$ represents the local attractor, respectively. *L* represents the characteristic length of the Delta potential well and hence, is the most important variable, because it determines the scope of the search being conducted. It is computed by $L=\frac{{\hbar }^2}{m\gamma }$, where m is the population size, $\hbar$ is the Laplace operator, and $\gamma$ is Plank’s constant. Further, the probability density function denoted by ${\psi }^2$ indicates the particles at one position relative to another and can be formulated by the following:

4 $$\begin{aligned} \left| \psi (y)\right| ^2=\frac{1}{{L}}e^{-2\left| y\right| / L } \end{aligned}$$

In order to procure the positions of each particle in the inference search space, the quantum state must first collapse to the classical state. The measurement of the process can be simulated by employing the Monte Carlo method using the probability density as its base. The mean best position is defined as the mean position of the personal best positions of all the particles in the population and is denoted by *mbest*. The *mbest* is then incorporated throughout the entire search process since it serves to balance the position diversity of all the particles and thus enhances the global search ability of QPSO. Then the value of *L* is computed by $2 \beta \left| {mbest}-X\right|$ by adopting the measuring method, where $\beta$ is a positive real number. Subsequent to QPSO’s performance of this measuring procedure, each particle’s position is accurately measured by

5 $$\begin{aligned} X=P_{id}\pm \frac{L}{2} \ln \left( \frac{1}{u} \right) \end{aligned}$$

where *u* is a random number uniformly distributed on the [0, 1] range.

It is important to emphasize that the evolution of all the particles is determined by considering each particle’s current position, i.e., the local best position and the global best position. For a discreate optimization problem with *d* parameters, we can represent a possible solution as a $d-$dimensional vector $X= \left( X_{1}, X_{2}, \ldots , X_{d} \right)$. Thus, given the QPSO with $d-$dimensional space, the current position of the *ith* particle is denoted by $X_{i}= \left( X_{i1}, X_{i2}, \ldots , X_{id} \right)$. The local best position of particle *i* is the best previous position (i.e., the position with the best fitness value), which is denoted by $Pbest_i= \left( Pbest_{i1}, Pbest_{i2}, \ldots , Pbest_{id} \right)$, and is called the personal best position (*Pbest*). The global best position is defined as the best position among all the particles in the population, called *gbest* and is denoted by $gbest_{i}= \left( {gbest}_{i1}, {gbest}_{i2}, \ldots , {gbest}_{id} \right)$. The value of *gbest* can be derived from the following equation: $gbest=min({pbest}_i)$.

During the evolution process, the position of each particle is iteratively updated generation by generation. The fundamental steps of QPSO for updating the current position of the evolution process of all particles are conducted as follows: first, the diversity of particles representing the mean best position is calculated; second, the local attractor of each particle as represented by the range of the search space is given; and finally, an update of the current position of each particle representing the candidate solution is completed. As described, *mbest* is defined as the mean personal best positions of the whole population and is given by

6 $$\begin{aligned} mbest=\frac{1}{M}\sum _{i=1}^M Pbest_i=\left( \frac{1}{M}\sum _{i=1}^M Pbest_{i1},\frac{1}{M}\sum _{i=1}^M Pbest_{i2}, \cdots ,\frac{1}{M}\sum _{i=1}^M Pbest_{id} \right) \end{aligned}$$

where *M* is the population size, and d is the dimension, respectively. The $P_{id}$ is defined as

7 $$\begin{aligned} P_{id}=\mu \cdot {Pbest}_i+(1-\mu )\cdot {gbest} \end{aligned}$$

where $P_{id}$ is the local attractor, and $\mu$ is a random number uniformly distributed in [0, 1], respectively. As indicated above, the main moving direction of the particles is $P_{id}$, which means that the space near $P_{id}$ is identified as a valuable searching area in QPSO. Finally, the position of each particle is given by

8 $$\begin{aligned} X_{id}=\begin{array}{cc} P_{id}\pm \beta \left| {mbest}-X_{id}\right| \ln \left( \frac{1}{u} \right) \end{array} \end{aligned}$$

where $X_{id}$ is the particle position, and *u* is a random number uniformly distributed on [0, 1], respectivley. Furthermore, $\beta$ is a contraction-expansion coefficient from 0.5 to 1.0, which can be tuned to control the convergence speed of the algorithm. As Sun suggested, a linear decrease in the value of $\beta$, namely, from 1.0 to 0.5, can result in a better convergence speed, which result is produced by the computation resulting from the following formula:

9 $$\begin{aligned} \beta = \frac{(1.0-0.5)\times (Maxiter-t)}{Maxiter} \end{aligned}$$

where *t* is the current generation, and *Maxiter* is the maximum generation, respectively. In addition, only one parameter, which is denoted by $\beta$, controls the position of particles; therefore, the parameters of QPSO are easy to control.

### An improved discrete QPSO algorithm

The QPSO is a metaheuristic algorithm that solves a problem by generating a population of candidate solutions (particles), which can further be optimized using iterative search. The QPSO algorithm with comparatively improved global search performance capabilities could overcome the problem of premature convergence that currently exists in the continuous optimization problems space. However, a problem that confounds the breadth of the performance potential of QPSO, is that it cannot be directly applied to discrete optimization problems. To overcome this shortcoming, Sun et al. proposed a binary QPSO algorithm (BQPSO) [26], which is characterized by a space transformation technique that is predicated on a binary coding scheme—that maps the consecutive searching space into a discrete searching space.

Although a wide range of applications have the characteristic of a discrete search space, the current literature we surveyed reflects less studies and analysis have been conducted on discrete optimization problems. Hence, this gap propelled us to discover a novel strategy to deal with such optimization problems. To overcome the problem that the data structure is not composed of sequences during the evolution process, an improved discrete QPSO algorithm (IDQPSO) is proposed in this paper. The proposed algorithm combines a novel strategy for updating the particles’ positions, thereby addressing the issue of the absence of a discrete sequence, which to-date, has hampered the effective deployment of QPSO.

#### Update by adopting two averages of the fitness value

**Fig. 3:**
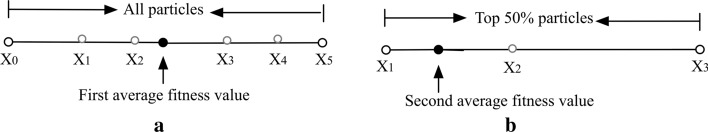
The procedure of updating *mbest* by adopting two averages of the fitness value. The left figure **a** represents the distance to the first average fitness value among all the particles, and $X_2$ is selected as *cbest* because it is the closest distance to the first average fitness value. The right figure **b** represents the distance to the second average fitness value among the top $50\%$ particles, and $X_1$ is selected as *mbest* because it is the closest distance to the second average fitness value

As described in QPSO, the mean best position, denoted by *mbest*, is defined as the center of the personal best positions among the whole population. In IDQPSO, *mbest* is obtained by averaging two of the computed fitness values and is inspired by the center of gravity in geometry. Here, the particle selected by applying two average operations of the fitness values, can be utilized to reflect the distribution of the whole population as well, since the first step of the IDQPSO algorithm comprehensively considers all of the particles, and thus, the second step can then improve upon the evolution process of the entire population. A concrete example of the procedure of updating *mbest* as redesigned in the IDQPSO algorithm, is illustrated in the Fig. 3. Figure 3a represents the distance to the first average fitness value among all the particles, and $X_2$ is selected as *cbest*. Figure 3b represents the distance to the second average fitness value among the top $50\%$ particles, and $X_1$ is selected as *mbest*. The procedure is detailed in the following portion of this paper.

To estimate the distance between two particles’ positions by their fitness values, we define that $dis(X_i,X_j)=\left| f(X_i)-f(X_j)\right|$, where $dis(X_i,X_j)$ represents the distance between particle $X_i$ and $X_j$, and the fitness value is denoted by *f*. First, to attain the initial selection of the candidate mean best position, a strategy of averaging the fitness values that represent the objective function value of the given personal best positions is proposed. Next, a particle with the closest distance to the average fitness value is selected as the candidate mean best position denoted by *cbest*. The *cbest*, as utilized by IDQPSO, is given by the following:

10 $$\begin{aligned} f(cbest) =\left( \frac{1}{M}\sum _{i=1}^M f(Pbest_{i1}),\frac{1}{M}\sum _{i=1}^M f(Pbest_{i2}), \ldots ,\right. \left. \frac{1}{M}\sum _{i=1}^M f(Pbest_{id}) \right) \end{aligned}$$

where *f*(*cbest*) is the first candidate mean fitness value, and $f(Pbest_{i})$ is the fitness value of the local best position, respectively.

Upon IDQPSO capturing the first average, the *cbest* representing the mean best position is obtained. However, we note that the final mean best position, denoted by *mbest*, can be further optimized by making another selection according to the averaging of fitness values. Although this objective function for producing an optimized selection process is similar to the first averaging strategy, it goes further and also computes the averages between the first selection of the candidate mean best position and the particles with better fitness values than *f*(*cbest*) (top $50\%$ particles). Thereafter, the particle with the closest distance to the second average fitness value is selected as the *mbest*.

To update *mbest* by averaging two selections of the average fitness values is reasonable from the perspective of causality, because this method also accounts for the search uncertainty and search probability within quantum mechanics. Furthermore, the updating strategy by applying two selections can serve to effectively balance the diversity of the evolution process in finding the best path and in avoiding premature convergence and fitness stagnation.

#### Evolving through generations

For discrete optimization problems, the evolution process is actually a comparison between positions. In general, since each element of the vector is independent, the swap operation is an effective strategy to employ in managing a discrete search space. For example, assume two positions $X_1=(1,2,3,4,5)$ and $X_2=(5,4,3,2,1)$, we can generate two new positions $X_1'$ and $X_2'$ by swapping the first element of these two vectors, resulting in $X_1'=(5,2,3,4,5)$ and $X_2'=(1,4,3,2,1)$. Let the fitness function be $f_i=\sum _{j=1}^d X_{ij}$. By checking all possible swaps, the new position (generated from the two parents $X_1$ and $X_2$) that gives the best fitness is $X_{best}=(5,4,3,4,5)$, by picking the larger value at each of the five element from the two parents.

It is obvious the above swap operation will not be valid in the median problem as the resulted new positions (genomes) may be invalid, thus we need a new strategy to conduct the evolution through generations. In this paper, we go further and take advantage of the whole-genome background in designing an efficient swap operation which is based on genomic sorting for use by the IDQPSO algorithm. Given two genomes (positions) $X_1$ and $X_2$, we generate a new genome $X_1'$ by sorting several DCJ events from $X_1$ to $X_2$, and $X_2'$ by sorting some step from $X_2$ to $X_1$. Since there may be multiple choices to make such DCJ sorting, we need to check every possibility and find the new genome that has the lowest fitness function, i.e., the median score. When the two genomes are large and distant, the computational cost, arising from the performance of the enumerating all sorting steps and computing median scores, rapidly increases due to the exhaustive searching strategy it employs. To overcome this problem, the IDQPSO algorithm incorporates an efficient search strategy which is detailed below.

### An IDQPSO-Median for ancestral genome inference

For complex problems and problems presenting high dimensional data, the global best position cannot be easily found. When the genomes are large and distant, the DCJ Median Problem is compounded by the additional obstacle presented by a challenge that has an ever increasing search space: assuming the length of genome is *n*, there are $2^nn!$ possible signed permutations [15]. As a consequence of the foregoing, the challenge of continuing the evolution process while avoiding premature convergence and escaping local optima is a critical and important procedure. It is necessary to maintain a population that allows each particle to evolve separately, while each generation keeps the whole population as a means to find the best fit.

Since the search space is so large that IDQPSO cannot converge to an optimum in a limited time without incorporating the sorting strategy proposed below. Consequently, in those circumstances, we propose that DCJ sorting be employed based on the established observation that the median genome is likely to be found on the path found during the evolution process in transforming one genome to another. Based on our combined previous experience, DCJ sorting is an effective evolution strategy for ancestral genome inference by reducing the search space. The detailed evolution process applied by the IDQPSO algorithm in finding the IDQPSO-Median is described as follows.

#### Algorithm overview

The IDQPSO-median maintains and updates three set of genomes, namely the current best median genomes $X_d=(X_{1d},\cdots ,X_{id}\cdots ,X_{Md})$, the intermediate best median genomes $P_d=(P_{1d},\cdots ,P_{id}\cdots ,P_{Md})$ and the personal best median genomes $P_{best}=(P_{best1},\cdots ,P_{besti}\cdots ,P_{bestM})$, each contains *M* genomes (*M* is the population size). We also maintain three best medians, i.e., *mbest* represents the mean best median from $P_{best}$, $P_{best}$ is the personal best median found so far from previous generations, and *gbest* is the generation best of $P_{best}$.

**Fig. 4:**
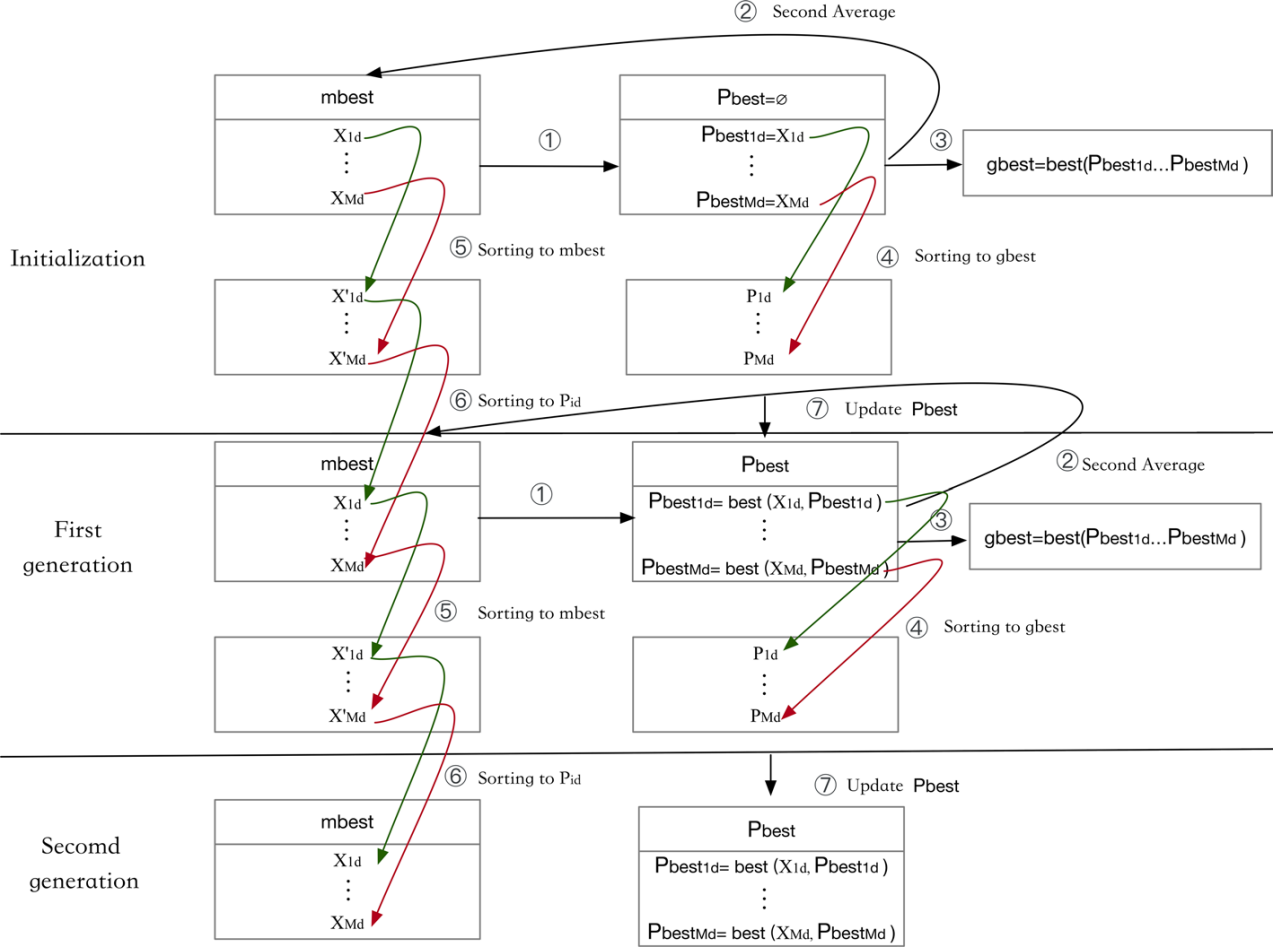
The illustration of algorithm overview. Assuming each generation contains a set of *M* genomes represented as $X_{1d}...X_{Md}$, as well as *mbest* and $P_{id}$. The population of the next generation is created by sorting each $X_{id}$ toward the best genome

We first initialize the *M* genomes in $X_d$ by using the method describe below and start the following steps (Fig. 4):

1. Update $P_{best}$: at the initialiation stage we simply assign $P_{besti}=X_i$; for all later generations, we assign $P_{besti}$ to be the one with lower median score from previous $P_{besti}$ and $X_{i}$;
2. Find the mean best *mbest* by averaging all median scores from $P_{best}$ and further average among top $50\%$ particles, *mbest* is the genome with its closest to the mean of the top $50\%$ scores;
3. Find the generation best *gbest* as the best (with the lowest median score) from $P_{best}$;
4. Update $P_{id}$ by sorting each genome in $P_{best}$ toward *gbest* if the later has better score;
5. Generate a new set $X_{id}'$ by sorting each genome in $X_{id}$ toward *mbest* if the later is better;
6. Create the next generation of candidate genomes $X_{id}$ by sorting each $X_{id}'$ toward its corresponding $P_{id}$ if the later is better;
7. Repeat the first step as the new generation until stop.

#### Initialization

Previous experience in this area foretells that the initialized candidate solution has a deep influence on both the computation cost and convergence speed. However, the search space within which the IDQPSO-Median must be located is too large, and thus any randomly selected genome is likely to generate candidates that deviate too much from the optimum. For this reason, if the IDQPSO algorithm used random selection to generate the initial candidates, the search process for finding the IDQPSO-Median could not converge to the optimum.

In order to expedite the evolution process of the particles without decreasing the performance of the IDQPSO algorithm, an initialization strategy of adopting DCJ sorting to reduce the search space has been integrated into the IDQPSO algorithm. First, DCJ sorting is applied to generate different candidate median genome sets along the evolutionary path from genome $G_i$ to genome $G_j$ which candidate median genomes are selected from the three given genomes $\{G_1,G_2,G_3\}$. In order to maintain the stochastic characteristic of the IDQPSO algorithm, six candidate median genome are selected with $\frac{1}{10}d_{DCJ} (G_i,G_j )$, $\frac{2}{10}d_{DCJ} (G_i,G_j )$, $\frac{3}{10}d_{DCJ} (G_i,G_j )$, $\frac{4}{10}d_{DCJ} (G_i,G_j )$, $\frac{5}{10}d_{DCJ} (G_i,G_j )$, and $\frac{6}{10} d_{DCJ} (G_i,G_j )$ corresponding steps away from $G_i$ to $G_j$.

Assuming the population size is *M*, there are six combinations for each set of two of the given genomes because of the direction of each genome in these two genome sets. After applying DCJ sorting, the total number of candidate median genomes is $M \times 6 \times 6$. Consequently, there are 36*M* candidate median genomes in the initial pool. After applying DCJ sorting, one is then randomly selected as the initial median genome from the first pool of candidate median genomes.

#### Fitness function

As a performance criterion to measure efficiency, the fitness value of a particle influences whether it is saved or not for the next generation. The fitness function is designed with the purpose of directing the evolutionary process for the entire population to continually improve the process of evolution until its cessation. For the DCJ Median Problem, setting the function of the median score as the fitness function is an efficient way to achieve the purpose encapsulated by the design of the fitness function. Since the median score represents how many DCJ operations have been conducted on the given genomes, setting it as the fitness function can result in the whole population evolving towards a better fit. The fitness function is given as follows:

11 $$\begin{aligned} F_G=d(G_1,G_m )+d(G_2,G_m )+d(G_3,G_m ) \end{aligned}$$

where $F_G$ represents the total DCJ distance of the three given genomes ${G_1}$, ${G_2}$, and ${G_3 }$, and where $G_m$ represents the median genome obtained as a result of the shortest sequence of DCJ operations on the three given genomes.

The fitness function indicates that the particle with a lower median score can have a better fitness value. Therefore, when compared to those with lower fitness values, those particles with better fitness values indicate an increased chance of surviving the evolution process.

#### Update the mean best median genome (*mbest*) by adopting two average of the median score

For ancestral genome inference, the initial position of each particle represents the initialized median genome. After the current median genome of all particles are set, the local best median genome, also called the personal best median genome (*Pbest*), can be obtained through an iterative comparison of the values of the previous best median genome to the current personal best median genome. For the complete set of particles in the inference solution search space, the median score between the given three genomes and the initialized median genome is calculated based on the fitness function. Then according to Eq (10), the first average median score denoted by *f*(*cbest*) is acquired. Next, by comparing the distance using $dis(X_i,X_j)=\left| f(X_i)-f(X_j)\right|$, the particle with the closest distance to the *f*(*cbest*) is selected as the first candidate median genome denoted by *cbest*.

Since the two averages of the median scores utilized to update the mean best median genome can reflect the distribution of a population, a further average between the first candidate median genome, *cbest*, and the particles with better median scores than *cbest* is captured and constitutes the average of the median scores. Similar to the initial average of the median scores, the particle that has the closest distance to the second average median score is extracted and is denoted by *mbest*. Finally, the *mbest* is selected as the mean best median genome for the ancestral genome inference.

#### Update the intermediate best median genomes ($P_{d}$) by adopting DCJ sorting

As the outset, it is important to emphasize that a reference to the intermediate best median genome is equivalent to the local attractor. The global best median genome can be found by comparing the intermediate best median genome with other particles according to the following formula: $gbest=min({pbest}_i)$. Consequently, the median genome with the lowest median score is set as *gbest*. Furthermore, the parameter $\mu {}$ is set to 0.5, which means the local best median genome and the global best median genome each assign the same weight to the intermediate best median genome.

In finding the IDQPSO-Median, DCJ sorting is applied to better guide and direct the inference process for inferring the optimal median genome. As the DCJ sorting path depends on the start and target genome, a target median genome that has a better median score than the start genome must be selected at the outset. Afterward, for the two selected genomes, in light of the accepted norm that the median score of the global best median genome will not be worse than the score of the local best median genome, the global best median genome is set as the target genome. By applying the DCJ sorting strategy, we sampled six candidate median genomes that fell on the DCJ sorting path with the following: $\frac{1}{10}d_{DCJ} (Pbest,pbest)$, $\frac{2}{10}d_{DCJ} (Pbest,gbest )$, $\frac{3}{10}d_{DCJ} (Pbest,gbest )$, $\frac{4}{10}d_{DCJ} (Pbest,gbest )$, $\frac{5}{10}d_{DCJ} (Pbest,gbest )$, $\frac{6}{10}d_{DCJ} (Pbest,gbest )$ steps away from *Pbest* to *gbest*. Then, we randomly selected one genome from these six candidates to constitute the intermediate best median genome. The results obtained indicate that the intermediate best median genome contains evolving material derived from its propagation of the local best median genome and global best median genome into the next generation, which results served to precipitate our articulation of the need for the overall search process to then proceed by conducting a further search based on the values found for the intermediate best median genome.

#### Update the current best median genomes ($X_{d}$) by adopting DCJ sorting

In order to deal with discrete gene orders, the parameter $\beta \times \ln \left( \frac{1}{u} \right)$ is set to 1. In IDQPSO-Median, the process for updating the current best median genome is comprised of two stages:

1. Generate $X_{id}'$: at each generation, there are *M* current best median genomes represented as $X_{1d},X_{2d},\cdots ,X_{id},\cdots ,X_{Md}$ and the mean best median as *mbest*. We will first compute the median score of $X_{1d},\cdots , X_{Md}$ and *mbest*, respectively. For each $X_{id}$, if *mbest* has better score, we then apply random steps of DCJ sorting from $X_{id}$ to *mbest* to get $X_{id}'$.
2. Obtain the next generation of $X_{id}$: the median score of all $X_{id}'$ and $P_{id}$ are computed. Assuming that $P_{id}$ has a better median score than $X_{id}'$, we then update the current best median genome $X_{id}$ by applying the random steps of DCJ sorting from $X_{id}'$ to $P_{id}$.

The process implemented in the IDQPSO algorithm for updating the current best median genome using a heuristic search strategy combined with the DCJ sorting strategy in order to avoid the problem of becoming trapped in a local optimum. Hence, we sampled a random number of steps away from the current best median genome towards the three given genomes, and then further randomly select one as the current median genome for the next generation.

As a result, each particle is required to update and further converge to an optimum that has the best median score. Once every particle has separately finished its respective evolution through generation, the evolution process of the whole population is completed. At this juncture, the global best median genome is finally considered to represent the most accurate and best option for the ancestral genome.

#### Pseudocode of the proposed IDQPSO-Median

In IDQPSO-Median, the maximum generations is set as *MaxIter*. The global median genome becomes closer and closer to the true ancestor through each stage of the evolution process. The process of updating terminates when the number of generations is satisfied. Otherwise, the updating process is repeated until the specified termination condition is reached. The details of the proposed IDQPSO-Median are shown in Algorithm1. 
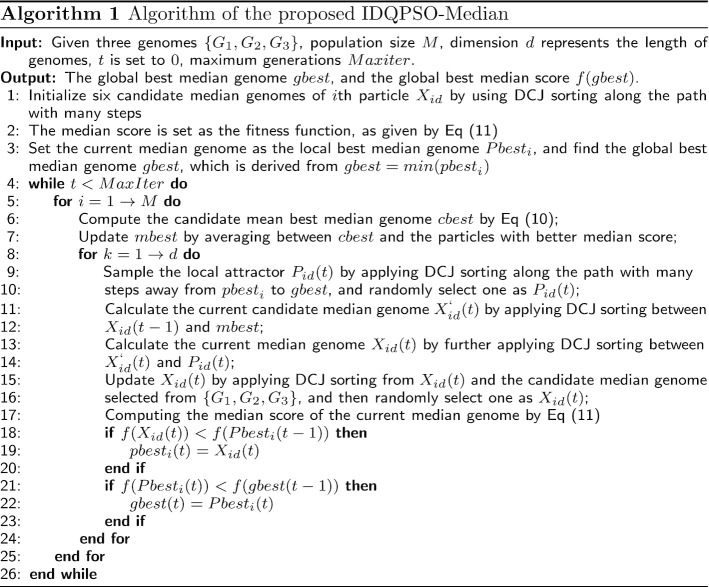


## Results

### Experimental environments and parameters setting

In order to evaluate the performance of IDQPSO-Median for phylogenetic reconstruction, we conducted extensive experiments in a variety of datasets using a spectrum of parameter settings therein. To obtain an unbiased CPU run time comparison, all of the experiments were run on a Dell PowerEdge R930 with Intel (R) Xeon (R) CPU E7-4820 v4*2 @ 2.10 GHz $*$ 20, 256 GB of Memory and 2048 GB of Disk space.

The primary parameter used as a performance benchmark was the number of genome rearrangement events per each edge represented on the generated adjacency graphs. Each genome has a different evolutionary rate, which evolutionary rates are given by $d=r/n$, where *r* represents the average number of rearrangement events along an edge and where *n* is the number of genes. Each dataset was comprised by three leaf nodes containing 1000 genes; the average number of reversal events per edge ranged from 100 to 1000. Consequently, the values that *d* took on ranged from 0.1 to 1. Twenty datasets were generated for each average number of reversal events.

SAMedian, GAMedian, and ASMedian were selected to test the effectiveness of the performance of the IDQPSO algorithm. The number of fitness function values (median scores) evaluated is considered representative of a fair time measurement in light of the deep insight into the convergence of the algorithms that the population size, the inner loops, and the number of generations provide. Therefore, every instance was executed with a set maximum number of function evaluations. The maximum number of sufficient generations of each median solver can provide that result in convergence was an important parameter at issue and was examined in the experiments we conducted. The parameters of all the median solvers were set to fall within the following bounds:

- IDQPSO-Median: the maximum generation and population size were set to be 2150 and 20, respectively, while every other parameter in IDQPSO-Median was set to one.
- SAMedian: the maximum generation was set to 10, 000, and the initial temperature and cooling rates were set to 10 and 0.9, respectively.
- GAMedian: the maximum generation was set to 100, and 50 genomes were generated for each step of sampling process.
- ASMedian: the parameters were set to the default values provided in the software package.

Given three genomes $G_1$, $G_2$ and $G_3$, the circular ordering lower bound is defined as $medianLB=(d_{12}+d_{13}+d_{23})/2$. To reduce the number of iterations of some easier cases, the search will stop if the best median score is equal to *medianLB*, or the number of iterations has exceeded either 2150 or $medianLB\times 1.5$.

### Performance of IDQPSO-Median

In our experiment, four different criteria (median score, distance to true ancestor, adjacency accuracy, and running time) were used to demonstrate the effectiveness of the IDQPSO-Median obtained by the approach implemented by the IDQPSO algorithm. The *median score* is defined as the sum of DCJ distances between each leaf genome and the inferred median. The *distance to true ancestor* is defined as the DCJ distance between the inferred median and the true ancestor genome. Given two sets of adjacencies, the median score value represents all of the adjacencies contained in the inferred median genomes, while the distance to true ancestor value represents the adjacencies from the true ancestor. In contrast, the *adjacency accuracy* is defined as the ratio of the intersection (denoted by $\cap$) of these two sets to the union (denoted by $\cup$) of these same two sets. The higher the value obtained for the adjacency accuracy, leads to the corresponding conclusion that the better the results the median solver returns will be. This relationship is given by the following:

12 $$\begin{aligned} Acc(G_m,G_t)=\frac{G_m \cap G_t}{G_m \cup G_t} \end{aligned}$$

where $Acc(G_m,G_t )$ represents the accuracy of adjacency, $G_m$ represents adjacencies in the inferred median genome, and $G_t$ represents the adjacencies in the true ancestor, respectively. The notation of $G_m \cap G_t$ represents the intersection of these two sets, $G_m$ and $G_t$, whereas, the notation of $G_m \cup G_t$ represents the union of these same two sets.

To highlight the performance of IDQPSO-Median, three groups of experiments were performed: wherein the population size was set as 20, 40, and 60, respectively. The results for the different population sizes examined in our experiments are set forth in Table 1.

**Table 1:** Performance of IDQPSO-Median with respect to size of population

# Events	Population size	Median score	Distance to true	Adj. accuracy	Mean time (s)
$r=100$	20	299.45	0.20	1	2
	40	299.45	0.20	1	2
	60	299.45	0.15	1	5
$r=200$	20	599.95	1.50	0.998	11
	40	599.95	1.05	0.999	15
	60	598.85	0.95	0.999	25
$r=300$	20	922.20	63.50	0.905	73
	40	921.15	62.05	0.909	81
	60	920.50	61.75	0.910	84
$r=400$	20	1244.35	251.85	0.684	89
	40	1242.25	250.80	0.686	91
	60	1240.45	249.50	0.687	124
$r=500$	20	1462.70	430.05	0.507	89
	40	1459.85	428.45	0.508	100
	60	1458.65	425.05	0.511	121
$r=600$	20	1610.50	574.60	0.367	62
	40	1608.25	572.85	0.370	87
	60	1608.80	572.75	0.370	140
$r=700$	20	1697.95	670.50	0.288	84
	40	1693.60	669.15	0.289	99
	60	1693.55	668.65	0.290	142
$r=800$	20	1763.70	748.85	0.214	95
	40	1759.85	748.15	0.216	125
	60	1759.35	747.85	0.218	155
$r=900$	20	1800.45	802.10	0.172	48
	40	1797.80	802.05	0.172	100
	60	1798.00	800.80	0.173	131
$r=1000$	20	1827.55	848.05	0.134	62
	40	1823.35	846.25	0.134	106
	60	1820.45	845.70	0.136	130

From the view of the population size, it can be seen that the values obtained by the IDQPSO algorithm for values of the median score, true distance, and adjacency accuracy became increasingly improved as the population size increased from 20 to 60. Although the improvements are relatively small, it does demonstrate that population size has some effect on the performance of our median solver. Furthermore, these results show that the performance of the IDQPSO algorithm in obtaining the IDQPSO-Median requires additional time to converge in the context of larger populations comprising the search space, and thus, incurs the attendant additional time cost as the population increases in size. For this reason, the larger the population size, the more expensive the time cost the IDQPSO algorithm will incur in finding the IDQPSO-Median. Moreover, our experimental results depict that the running time did not increase dramatically as the number of the rearrangement events grew. Taking the analysis above into account, the population size was finally set as 60 for comparative purposes since the IDQPSO algorithm can achieve a relatively competitive performance with less computation cost when compared to the other median solvers.

### Comparison with SAMedian, GAMedian and ASMedian

#### Median score

Lower median scores are desired and sought to be returned, and thus, the lower the values for the median score obtained by one of the tested models, the better the performance it demonstrated in our experiments. The comparison of IDQPSO-Median, SAMedian, GAMedian, and ASMedian are provided in Table 2, and a detailed description of our experimental results is also described below. As shown in Table 2, when the number of rearrangement events ranges from 600 to 1000, the IDQPSO-Median achieves the best median scores as measured against the results obtained by all of the parsimony-based methods, whereas ASMedian obtains better median scores when the number of rearrangement events is smaller than 600. However, despite that ASMedian performs relatively worse if compared with the IDQPSO-Median approach when the number of rearrangement events is more than 600, ASMedian can still obtain better optimums in the context of different rearrangement events when compared to the results obtained by GAMedian and SAMedian. Furthermore, the differences in the median scores themselves obtained by the models we tested are relatively apparent, and GAMedian has the worst performance. An analysis of these results between IDQPSO-Median and ASMedian reveals that IDQPSO-Median has a better capability for solving complex high-dimensional problems versus ASMedian when the genomes are large and distant.

**Table 2:** Median score of IDQPSO-Median, SAMedian, GAMedian, and ASMedian

Median solver	Median score									
	r = 100	r = 200	r = 300	r = 400	r = 500	r = 600	r = 700	r = 800	r = 900	r = 1000
IDQPSO-Median	*299*.*5*	598.9	920.5	1240.5	*1458.7*	*1608*.*8*	*1693*.*6*	*1759*.*4*	*1798*.*0*	*1820*.*5*
SAMedian	299.5	599.4	933.4	1284.4	1516.1	1664.0	1750.3	1811.4	1850.0	1876.2
GAMedian	333.0	747.8	1166.6	1464.1	1648.5	1764.8	1835.3	1890.4	1918.3	1940.0
ASMedian	299.5	*598*.*8*	*898*.*4*	*1227*.*3*	*1460*.*7*	1621.8	1719.3	1790.9	1830.2	1856.2

The best values of all the compared algorithms are indicated in italics

#### Distance from median genome to actual ancestor

One indicator of the quality of the inferred median genome is the distance between the inferred median genome and the true ancestor. The observed distances to true ancestors attained by the four median solvers in the context of differing rearrangement events are displayed in Table 3. Our results show that our IDQPSO-Median achieves the best performance and also obtains the lowest distance to the true ancestor when the number of rearrangement events are $\ge 500$, as well as when the number of events is 100. As shown in Table 3, ASMedian can obtain the lowest distance provided that the number of rearrangement events is smaller than 500, while our IDQPSO-Median is the second best-performing algorithm producing only relatively worse results than the best method. It is described in Table 3, these experimental results show that the other median solvers outperform other metaheuristic methods such as GAMedian and SAMedian under different rearrangement events. By analyzing these results, it is evident that our IDQPSO-Median performs relatively better when the genomes are distant and achieves similar performance in the context of other genome arrangement events.

**Table 3:** Distance to true ancestors of IDQPSO-Median, SAMedian, GAMedian, and ASMedian

Median solver	Distance to true ancestors									
	r = 100	r = 200	r = 300	r = 400	r = 500	r = 600	r = 700	r = 800	r = 900	r = 1000
IDQPSO-Median	*0*.*15*	0.95	61.75	249.50	*425.05*	*572*.*75*	*668*.*65*	*747*.*85*	*800*.*80*	*845*.*70*
SAMedian	0.25	1.75	75.65	290.25	467.20	602.70	699.40	767.00	817.60	857.90
GAMedian	30.35	147.40	284.65	389.40	500.95	595.50	677.45	754.20	803.60	848.40
ASMedian	0.35	*0*.*85*	*12*.*20*	*241*.*05*	451.35	615.90	726.40	802.55	854.40	888.00

The best values of all the compared algorithms are indicated in italics

#### Adjacency accuracy

Adjacency accuracy is defined as the proportion between the inferred median genome and the true ancestor of the intersection of their adjacencies to the union of their adjacencies. The accuracy of the adjacency values obtained by the selected median solvers are depicted in Table 4. From these results, it is clear that when the number of rearrangement events is $\ge 400$, the IDQPSO-Median approach obtains the best values for adjacency accuracy, while when the number of rearrangement events is less than 400, the adjacency accuracy values found by the approach of ASMedian is the highest and thereby returns the most accurate values. By analyzing these results, we reach the conclusion that the IDQPSO-Median approach returns the highest values of adjacency accuracy when the number of rearrangement events becomes larger, and we can also derive from our results that the accuracy of adjacency of IDQPSO-Median becomes lower and thereafter, continues to decrease as the number of rearrangement events increase. In summary, the IDQPSO-Median approach indicates its competitive performance in obtaining good values for the accuracy of adjacency when compared with the other median solvers analyzed in this experiment—SAMedian, GAMedian, and ASMedian.

**Table 4:** Adjacency accuracy of IDQPSO-Median, SAMedian, GAMedian, and ASMedian

Median solver	Adjacency accuracy									
	r = 100	r = 200	r = 300	r = 400	r = 500	r = 600	r = 700	r = 800	r = 900	r = 1000
IDQPSO-Median	*1*.*00*	0.999	0.910	*0*.*687*	*0*.*511*	*0*.*370*	*0*.*290*	*0*.*218*	*0*.*173*	*0*.*136*
SAMedian	1.00	0.99	0.80	0.48	0.31	0.208	0.149	0.112	0.086	0.066
GAMedian	0.89	0.60	0.40	0.31	0.24	0.184	0.147	0.112	0.089	0.071
ASMedian	1.00	*1*.*00*	*0*.*96*	0.57	0.35	0.219	0.146	0.101	0.073	0.055

The best values of all the compared algorithms are indicated in italics

#### Running time

Computational cost is an important performance criterion. The running time of IDQPSO-Median is largely determined by the time spent on generations during evolution and only an effective CPU time is considered in our experiments. As shown in Table 5, the running time of GAMedian is relatively more expensive than all the other median solvers we analyzed when the number of rearrangement events was less than 600. Besides, the running time of ASMedian increases dramatically when the number of rearrangement events is more than 600. Based on these experimental results, the total running time of ASMedian is approximately 40 h when the number of rearrangement events is 1000. In addition, the time cost of GAMedian is considerably expensive. In specific terms, the running time of GAMedian for each generation is 330 seconds, namely, the total running time is approximately 92 h. As a result, the total running time of 20 genomes is about 75 days when the maximum generation is set with a limit of 1000.

**Table 5:** Mean running time of IDQPSO-Median, SAMedian, GAMedian, and ASMedian

Median solver	Mean running time (s)									
	r = 100	r = 200	r = 300	r = 400	r = 500	r = 600	r = 700	r = 800	r = 900	r = 1000
IDQPSO-Median	5	25	*84*	*124*	*121*	*140*	*142*	*155*	*131*	*130*
SAMedian	277	327	430	470	503	454	440	427	422	417
GAMedian	36,112	34,779	34,091	33,436	32,994	32,908	327,35	32,528	32,445	32,420
ASMedian	*1*	*1*	2725	12,875	17,787	48,625	96,020	123,077	131,510	142,356

The best values of all the compared algorithms are indicated in italics

In this experiment, IDQPSO-Median is the fastest except for the simplest datasets, where ASMedian needs about a second to finish. Compared to other two heuristics, IDQPSO-Median is not only much faster, it also has a better performance than SAMedian on the median score, true distance, and adjacency accuracy when the genomes are large and distant.

### Phylogeny reconstruction and ancestor inference

We integrated the new median solver with the GRAPPA framework which utilizes an iterative approach to score a tree. To find the best tree with its associated internal (ancestral) genomes, enumerates and scores all possible tree topologies using an iterative approach. For a tree T for which both the leaf genomes and internal genomes are known, we can easily compute the weight of each edge and the tree score *w* is defined as summing all the edge lengths. However, since the internal genomes are unknown at first, the task of scoring a tree is to find the best assignment of gene orders on the internal nodes that gives the lowest tree score.

The GRAPPA scoring procedure has two stages: initialization and iteratively update. For two nodes in a tree, we define the path length as the number of edges in the shortest path from one to another. For each internal genome, we define a median problem using three leaf (known) genomes that have the shortest path length to it. GRAPPA can then solve this median problem and use the resulting median as the initial gene order for this internal node. GRAPPA then iteratively solves the median problem on each internal node using a depth-first approach and updates if a better genome is found until no change occurs. The tree score is finally computed by summing all edge lengths based on the final genomes assigned on the internal nodes.

It is obvious that such iterative procedure is expensive as it needs to solve numerous median problems. To overcome this problem, GRAPPA uses the circular ordering lower bound to eliminate most trees that are not worthy of being scored. The lower bound is based on the following observation. Given *n* genomes, let $d_{i,j}$ be the pairwise distance between genomes *i* and *j*. Given a tree T and its score *w*(*T*), if $1,2,\cdots ,n$ is a circular ordering of the leaves of T, then we have $2w(T)\ge d_{1,2}+d_{2,3}+\cdots +d_{n,1}$ based on the triangular inequality. In other words, if the best tree so far has score $w_{best}$ and $d_{1,2}+d_{2,3}+\cdots +d_{n,1}> 2w_{best}$, the score of T must be larger than $w_{best}$, thus it can be safely discarded. To utilize this lower bound, the GRAPPA framework first computes the neighbor joining tree and uses its score as the best-so-far. It then enumerates all possible trees and updates the best-so-far when a better tree is found, pruning trees that have the lower bound larger than the best-so-far. The speed of GRAPPA relies on fast and accurate median solvers: it not only needs to quickly compute many instances of the median problem, but also needs to find as lower possible tree scores to tighten the lower bound with smaller best-so-far scores. To balance the speed and accuracy, in our experiments, we set the population size to be 20, and the maximum number of generations to be 500.

We conducted experiments using both simulated and real datasets. To generate simulated datasets, we randomly create tree topologies with 12 leaves and each genome has 1000 genes. We set the expected number of events along each edge to be $r = 20$–180. For each edge with the expected length *r*, the actual edge length is uniformly sampled between 0.1*r* and 1.9*r*. We use two combinations of types of events: one with only inversion, and one with 90% inversion and 10% transposition. We then assign the identity genome to the root, and populate each node with respect to the number of events along the path.

We compare our new method with both GRAPPA 2.0 (using an exact median solver) and SA-GRAPPA using SAMedian as the solver. In our experiments, GRAPPA 2.0 cannot finish scoring even the neighbor joining tree for $r\ge 100$ (for datasets with transpositions, GRAPPA failed at $r\ge 80$), thus its result on those datasets are not recorded. For $r\ge 140$, as the distances between genome pairs are large, the edit distance becomes seriously under estimate the true distance, making lower bound loose and many trees have to be scored. For these datasets, we use a different approach by sorting trees with respect to their lower bound and compute those with smaller lower bound first, with the assumption that better trees have smaller lower bound. We report results based on the best tree found after 5 days of computation.

An edge in the inferred tree is false positive (FP) if it is missing in the true tree. Similarly, an edge in the true tree is false negative (FN) if it is missing in the inferred tree. For *n* leaves, the Robinson–Foulds (RF) error rate is defined as $RF=(FP+FN)/2(n-2)\times 100\%$. Table 6 shows the RF error rate. For $r\le 80$, all methods (including neighbor joining) are very accurate and return trees without error. However, for more difficult trees, the neighbor joining method becomes less accurate with 20–$30\%$ errors. IDQPSO is still the most accurate with error rate $<5\%$ for datasets without transposition and $<10\%$ for datasets with transposition. SAMedian is less accurate and GRAPPA cannot finish any tree.

**Table 6:** Average Robinson–Foulds (RF) errors for IDQPSO, Simulated Annealing and GRAPPA

Program	RF error (%) (No transposition)								
	r = 20	r = 40	r = 60	r = 80	r = 100	r = 120	r = 140	r = 160	r = 180
IDQPSO-Median	0	0	0	*0*	*0*	*0*	*2*.*5*	*2*.*5*	*3*.*8*
SA-Median	0	0	0	2.5	0	2.5	6.3	5.0	10.0
GRAPPA-Exact	0	0	0	*0*	–	–	–	–	–
Neighbor-joining	0	0	0	2.5	6.3	6.3	12.5	20	20

Program	RF error (%) (90% Inversion/10% transposition)								
	r = 20	r = 40	r = 60	r = 80	r = 100	r = 120	r = 140	r = 160	r = 180
IDQPSO-Median	0	0	0	0	0	*2*.*5*	*2*.*5*	*2*.*5*	*8*.*8*
SA-Median	0	0	0	0	0	*2*.*5*	3.8	3.8	12.5
GRAPPA-Exact	0	0	0	–	–	–	–	–	–
Neighbor-joining	0	0	0	0	3.8	13.8	16.3	23.8	30

– indicates a program cannot finish after 5 days of computation. For the IDQPSO and Simulated Annealing methods, results for $r\ge 140$ are from the best trees obtained within 5 days of computation

The best values of all the compared algorithms are indicated in italics

Table 7 shows the scores of the reconstructed phylogenetic trees. For easier datasets ($r\le 80$), both IDQPSO and GRAPPA return the same best tree scores, although IDQPSO is a bit slower (Table 8). For more difficult datasets, IDQPSO is much faster than the SAMedian (Table 8) and dominates the performance of tree scores by finding trees with fewer number of events.

**Table 7:** Average score of the best tree for IDQPSO, Simulated Annealing and GRAPPA

Program	Tree score (No transposition)								
	r = 20	r = 40	r = 60	r = 80	r = 100	r = 120	r = 140	r = 160	r = 180
IDQPSO-Median	496.9	*985*.*7*	*1458*.*7*	*1847*.*3*	*2069*.*8*	*2730*.*7*	*3601*.*1*	*4092*.*6*	*4953*.*0*
SA-Median	496.9	986.0	1459.9	1862.3	2086.4	2792.6	3705.7	4306.1	5210.9
GRAPPA-Exact	496.9	*985*.*7*	*1458*.*7*	*1847*.*3*	–	–	–	–	–

Program	Tree score (10% inversions/10% transpositions)								
	r = 20	r = 40	r = 60	r = 80	r = 100	r = 120	r = 140	r = 160	r = 180
IDQPSO-Median	*526*.*6*	*1033*.*0*	1611.3	*2158*.*6*	*2448*.*7*	*3363*.*8*	*4435*.*0*	*4903*.*7*	*5078*.*4*
SA-Median	527.4	1033.6	1614.2	2174.5	2473.3	3447.1	4643.2	5070.0	5352.4
GRAPPA-Exact	527.4	1033.6	*1610*.*3*	–	–	–	–	–	–

– indicates a program cannot finish the scoring of any tree after 5 days of computation. For the IDQPSO and Simulated Annealing methods, results for $r\ge 140$ are from the best trees obtained within 5 days of computation

The best values of all the compared algorithms are indicated in italics

Table 9 shows the accuracy of the inferred ancestors of the tree by using the DCJ distance between the genome at the root of the tree with the identity genome which is used to generate the simuated datasets. All three methods inferred the ancestor that is identical to the true ancestor for easier datasets ($r\le 60$) while IDQPSO remains very accurate up to $r = 120$. For datasets without tranpositions, even for difficult datasets such as $r=140$ and $r=160$, the ancestors inferred by IDQPSO is only a few DCJ events away from true ancestors. The accuracy decreases when 10% transpositions are added, but remains below 100 for the most difficult datasets.

**Table 8:** Average running time for IDQPSO, Simulated Annealing and GRAPPA

Program	Running time (s) (No transposition)								
	r = 20	r = 40	r = 60	r = 80	r = 100	r = 120	r = 140	r = 160	r = 180
IDQPSO-Median	118.1	138.7	178.0	*295*.*6*	*835*.*9*	*19,839*.*4*	$>7$ days	$>7$ days	$>7$ days
SA-Median	167.4	298.6	577.1	1361.7	7930.5	42,249.7	$>7$ days	$>7$ days	$>7$ days
GRAPPA-Exact	*107*.*3*	*109*.*1*	*122*.*4*	365.3	–	–	–	–	–

Program	Running time (s) (90% inversions/10% transpositions)								
	r = 20	r = 40	r = 60	r = 80	r = 100	r = 120	r = 140	r = 160	r = 180
IDQPSO-Median	114.9	135.4	*238*.*3*	*1096*.*3*	*6556*.*1*	*32,388*.*5*	$>7$ days	$>7$ days	$>7$ days
SA-Median	155.6	398.7	746.7	7429.0	26,607.9	164,978.2	$>7$ days	$>7$ days	$>7$ days
GRAPPA-Exact	*113*.*0*	*113*.*9*	*4158*.*3*	–	–	–	–	–	–

For $r\ge 140$, both IDQPSO and Simulated Annealing are stopped after 5 days of computation

The best values of all the compared algorithms are indicated in italics

**Fig. 5:**
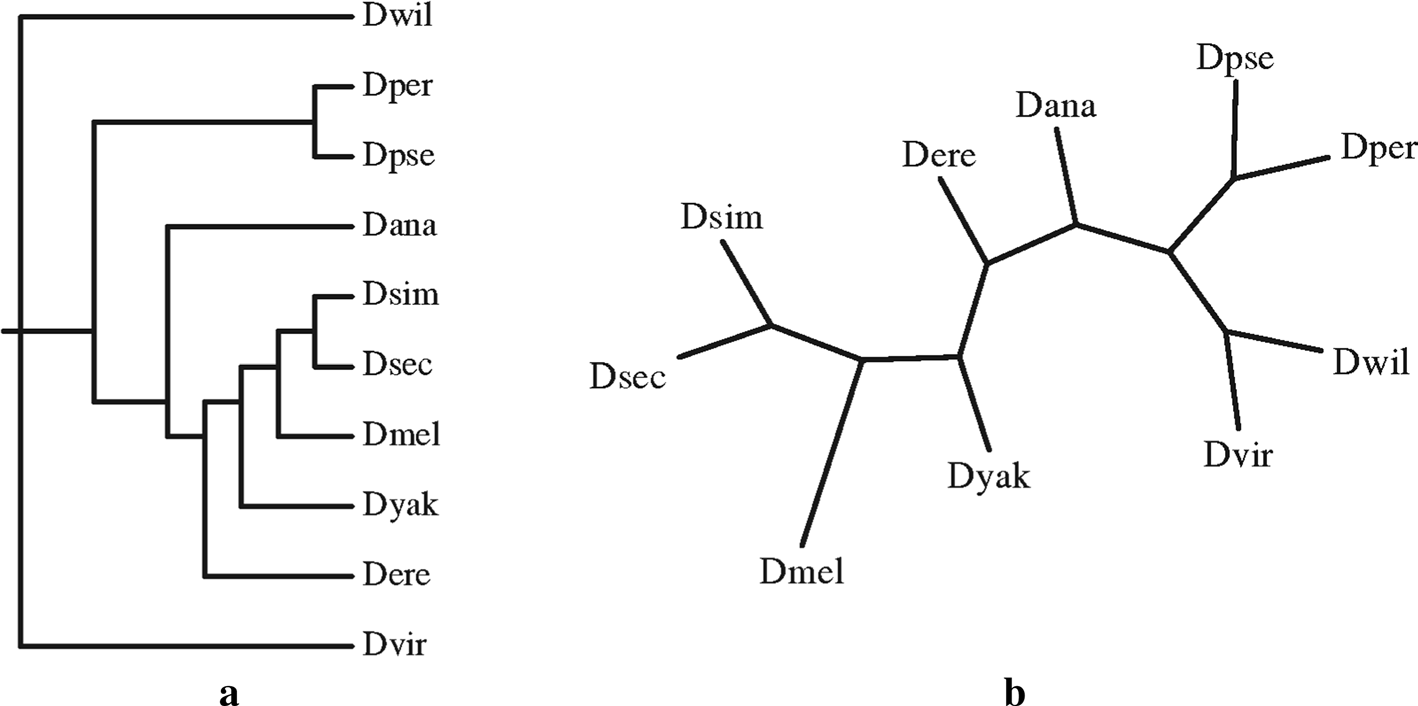
The topology of species. The left figure **a** shows the true topology of 10 drosophila species, the right figure **b** shows the inferred topology by the QPSO-GRAPPA method

We also conduct experiments on some biological datasets including the genomes of 10 drosophila species, each with 7332 genes. Fig. 5 shows both the phylogeny published by Clark et al. [27] and the tree reconstructed by IDQPSO, which demonstrates that our new method is able to infer accurate phylogenies for large genomes in real data.

**Table 9:** Average distance between the inferred and true tree ancestors for IDQPSO, Simulated Annealing and GRAPPA

Program	Distance to the true ancestor (No transposition)								
	r = 20	r = 40	r = 60	r = 80	r = 100	r = 120	r = 140	r = 160	r = 180
IDQPSO-Median	0	0	0	*0*	*0*.*2*	*0*.*3*	*5*.*2*	*7*.*7*	*43*.*8*
SA-Median	0	0	0	0.8	1.9	2.0	7.3	27.8	55.0
GRAPPA-Exact	0	0	0	*0*	–	–	–	–	–

Program	Distance to the true ancestor (90% inversions/10% transpositions)								
	r = 20	r = 40	r = 60	r = 80	r = 100	r = 120	r = 140	r = 160	r = 180
IDQPSO-Median	0	0.2	0	*1*.*7*	*2*.*0*	*10*.*1*	*47*.*7*	*53*.*0*	*99*.*6*
SA-Median	0	0.2	0	2.1	3.1	13.9	69.8	60.6	115.4
GRAPPA-Exact	0	0.2	0	–	–	–	–	–	–

– indicates a program cannot finish the scoring of any tree after 5 days of computation. For the IDQPSO and Simulated Annealing methods, results for $r\ge 140$ are from the best trees obtained within 5 days of computation

The best values of all the compared algorithms are indicated in italics

## Discussion

Based on the experimental results set forth above, a conclusion can be reached that all of the compared median solvers can have their own different advantages and disadvantages. The search space of the median problem is extremely large when the genomes are distant and large, which presents a crucial task that continues to challenge the available techniques in this area. However, GAMedian is quite limited by its lower speed and its low potential for scalability arising from the necessity of it maintaining a large candidate pool in order to obtain the optimal solution. In comparison, SAMedian achieves the second best performance on performance indicator of running time; however, these performance results come at significant disadvantages insofar as it returns lower performance scores on three of the most significant criteria–namely, the median score, true distance, and adjacency accuracy scores–which presents a palpable limitation on its practical applications. Moreover, ASMedian further requires a considerable amount of storage space and comprises a heavy burden for RAM since most of these partial solutions are saved onto the hard disk.

In comparison with the other median solvers, when the genomes are large and distant, IDQPSO-Median can achieve the relatively best scores for the performance indicators of the median score, the true distance score, and the score for measuring the adjacency accuracy. Because QPSO has fewer parameters to control, it requires the capacity to iteratively search to find the global optimal and local optimum. In contrast, IDQPSO-Median strategically preserves a piece of useful heuristic information from the current generation to the next and thus, consequently shortens the number of generations to reach convergence. Furthermore, the number of rearrangement events has no effect on the computation cost, which means the IDQPSO-Median approach can overcome the premature convergence problem even while the number of rearrangement events continues to increase. In conclusion, the IDQPSO-Median approach we propose has a superior performance when considering each of the significant criteria used in measuring the performance of median solvers for ancestral genome inference, including the performance metric of its running time.

In the experiments, it can be found that IDQPSO-Median, SAMedian, and GAMedian can sustain their performance levels at a consistent speed even when the rearrangement events become larger, which means metaheuristic algorithms can solve the complexity problem presented by genome rearrangement problems notwithstanding that these problem arise in the context of large and distant genomes. When compared to the other criteria we have used to evaluate the approaches we tested, an additional consequential factor, as compared to the other criteria, is whether it can converge with fewer generations or can converge even if it does not incorporate the use of computational algorithms. GAMedian is far too expensive to face this set of challenges, for this reason, the generation maximum for GAMedian was set as 100.

In contrast, the performance of the IDQPSO-Median demonstrates that IDQPSO-Median has better scalability when compared with the existing parsimony-based methods. Therefore, the approach proposed in our IDQPSO-Median is significant in light of the performance metrics achieved by the other state-of-the-art methods since it can currently provide better scalability for phylogenetic reconstruction when the genomes are large and distant. Based on the results of our experiments and the analysis set forth above, the advantages obtained by the IDQPSO-Median approach we propose not only rivals the advantages derived from the methods implemented by the other median solvers we compared but also sets a new competitive paradigm for use in ancestral genome inference.

## Conclusions

This paper has proposed an IDQPSO-Median for ancestral genome inference. We first propose the IDQPSO algorithm that adopts a process of obtaining two averages of the fitness values in order to find the mean best position and then utilizes the sorting operation to realize the evolution of IDQPSO. Next, we introduced an IDQPSO-Median, which incorporates DCJ sorting into the IDQPSO algorithm, for undertaking ancestral genome inference. We also conducted experimental studies that have served to demonstrate the effectiveness of the IDQPSO-Median approach over comparable median solver methods—ASMedian, SAMedian, and GAMedian. When the genomes are large and distant, the IDQPSO-Median obtains the lowest median score, the highest adjacency accuracy, and the closest distance to the true ancestors. In conclusion, our IDQPSO-Median achieves exceptional prowess in its flexibility, which results in its better scalability, and does so, even when faced with increasing whole-genome data. Thus, the IDQPSO algorithm and its incorporation of the IDQPSO-Median approach can effectively be applied to address the critical median problem that continues to challenge the current state-of-the-art techniques used in ancestral genome inference.

Although IDQPSO-Median has a desirable performance, especially when the genomes are large and distant, a few issues exist that warrant our further attention and study. In this vein, we would like to extend our work within larger genome rearrangement events, such as deletion, insertion, and duplication. In addition, we could finish all of the experiments we conduct on the quantum computer in order to increase the computation speed by way of using the characteristics of the quantum algorithm: because the quantum computer is times faster than any classical computer. Moreover, we would like the opportunity to take advantage of distributed computation capacity using Spark, which is a distributed framework that is designed to expedite computation. Finally, this research shows us that we have the potential to find deep evolutionary histories using deep learning algorithms if more datasets with unequal gene length are supplied.

## Data Availability

An C code implementation of the method is freely available at: https://github.com/jennifer19/median_solver.

## Abbreviations

IDQPSO: An improved discrete quantum-behaved particle swarm optimization algorithm
IDQPSO-Median: A median solver by incorporating the DCJ sorting into IDQPSO
ASMedian: Adequate subgraphs median
GAMedian: GA-based median
SAMedian: Simulated annealing median
QPSO: Quantum-behaved particle swarm optimization
GA: Genetic algorithm
